# Ghrelin protects against nucleus pulposus degeneration through inhibition of NF-κB signaling pathway and activation of Akt signaling pathway

**DOI:** 10.18632/oncotarget.19695

**Published:** 2017-07-31

**Authors:** Weiwei Li, Xihai Wu, Ruize Qu, Wenhan Wang, Xiaomin Chen, Lei Cheng, Yaoge Liu, Linlin Guo, Yunpeng Zhao, Chao Liu

**Affiliations:** ^1^ Department of Pathology, Qilu Hospital, Shandong University, Jinan, Shandong 250012, P. R. China; ^2^ Department of Gynaecology and Obstetrics, Jinan Central Hospital, Shandong University, Jinan, Shandong 250012, P. R. China; ^3^ Medical School of Shandong University, Jinan, Shandong 250012, P. R. China; ^4^ Department of Orthopaedics, Qilu Hospital, Shandong University, Jinan, Shandong 250012, P. R. China; ^5^ Department of Oral and Maxillofacial Surgery and Institute of Dental Medicine, Qilu Hospital, Shandong University, Jinan, Shandong 250012, P. R. China

**Keywords:** nucleus pulposus, IL-1β, ghrelin, NF-κB signaling, Akt signaling

## Abstract

The objective of the present study was to examine the potential role of ghrelin in degeneration of nucleus pulposus (NP). Lower expression levels of ghrelin were found in human NP cells stimulated with interleukin-1β (IL-1β). Moreover, exogenous ghrelin suppressed IL-1β induced degeneration and inflammation associated biomarkers in human NP cells, including matrix metalloproteinase-13, a disintegrin and metalloproteinase with thrombospondin motifs-5, tumor necrosis factor-α and iNOS, which was possibly mediated by antagonization of NF-κB signaling. Moreover, ghrelin enhanced production of critical extracellular matrix of NP cells, including collagen 2, aggrecan, and Sox-9 in NP cells. Ghrelin also promoted NP tissue regeneration in a rabbit IVD degeneration model, which seems to be associated with growth hormone secretagogue receptor. Additionally, the protective role of ghrelin in anabolism potentially relies on activation of Akt signaling pathway. Taken together, ghrelin may represent a molecular target for prevention and treatment of intervertebral disc degeneration.

## INTRODUCTION

Disc degeneration, a major cause of lower back pain, is characterized by the loss of water-binding proteoglycans that occurs through increased degradation and an overall shift toward a more fibrotic matrix in nucleus pulposus [1]. The resulting dehydration causes a reduction in disc height and an altered mechanical loading that leads to progressive changes in tissue microenvironment and cell function [2].

A characteristic change of IVD during degeneration is the decreased anabolism of NP cells [3]. It is well accepted that collagen 2 and aggrecan are key structural components in cartilage including NP tissue, and reduced production of these matrix molecules implies exaggerated degeneration in IVD [4]. Various studies have reported that enhancement of anabolism in NP cells may serve as a potential target for protection from IVD degeneration [5]. It is also well known that exaggerated catabolism occurs during IVD degeneration, while IL-1β has negative impact on this process [6]. IL-1β drives the expression of many degenerative matrix metalloproteinases (MMPs) and aggrecanases, such as A disintegrin and metalloproteinase with thrombospondin motifs 5 (ADAMTS-5) and MMP-13 [7–9]. Furthermore, IL-1β leads to enhanced production of other inflammatory molecules, resulting in atypical proliferation, cell death, synthesis of a fibrotic matrix, and more severe degeneration in IVD tissue, while inhibition of IL-1β signaling pathways leads to decreased IVD degeneration [6].

Ghrelin is a multifunctional peptide, that has initially been detected in gastrointestinal system [10]. Ghrelin has an essential role in various physiological and disease processes such as gastric ulcer, tumorogenesis, tissue repair and chondrogenesis [11–13]. It has been reported that interaction between ghrelin and its receptor, growth hormone secretagouge receptor (GHSR) [14], is vital for the effectiveness of ghrelin in many different processes. In recent years, different groups have identified the anti-inflammatory function of ghrelin [15]. Besides, ghrelin has been found to attenuate inflammatory bowel disease [16]. Ghrelin has also been associated with IL-1β, a critical cytokine in degeneration of IVD [17]. Furthermore, IL-1β induces stimulation of NF-κB signaling pathway, which aggravates IVD degeneration [18]. In addition, it is known that Akt signaling pathway plays a critical role in anabolism of NP cells [19]. Ghrelin is reported to antagonize NF-κB signaling pathway and activate Akt signaling pathway in several diseases [20, 21]. In the present study, we examined the expression pattern and potential role of ghrelin in degeneration of NP cells, and investigated whether NF-κB and Akt signaling pathways are involved in the protective function of ghrelin.

## RESULTS

### Ghrelin is detected in NP tissue and IL-1β decreases expression level of ghrelin in NP cells

**Figure 1:**
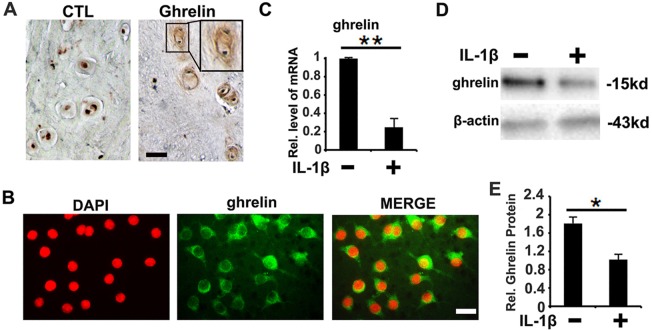
Expression pattern of ghrelin in NP cells **(A)** Ghrelin is detected in the extracellular matrix of the cell clusters formed in NP tissue from degenerated discs, as measured by immunohistochemistry. Left panel is negative control. Samples from disc degeneration patients were collected and were stained with anti-ghrelin antibody (brown), and consequently counterstained with methyl green (green). The insert indicates the higher magnification view of cell clusters. Scale bar, 25μm. **(B)** Ghrelin is expressed in plasma of primary human NP cells, as detected by cell immunostaining approach. NP cells were isolated, and cell immunostaining was performed for ghrelin. **(C)** RNA level of ghrelin was reduced by IL-1β, as assessed by real time PCR. **(D, E)** Protein level of ghrelin is reduced post- IL-1β treatment, as assayed by Western blot. The values are the mean±SD. ^*^p < 0.05 and ^**^ p < 0.01 vs. Control group. Scale bar=25μm. Each experiment was run in triplicate.

To date, it is unknown whether ghrelin is expressed in NP tissue of IVD. To investigate the expression pattern of ghrelin, NP tissue was collected from patients with degenerative disc disease (DDD) undergoing discectomy. Immunohistochemistry data showed the presence of ghrelin in NP tissue, mainly the extracellular matrix of NP cells (Figure 1A). Furthermore, the presence of ghrelin was examined at the cellular level using immunostaining approach. As shown in Figure 1B and Supplementary Figure 1A, ghrelin expression was detected in NP cells, especially in cytoplasm. IL-1β is well known factor in degeneration of NP tissue and has become a potential target for treatment of DDD. In order to investigate the association between ghrelin and IL-1β in NP, human NP cells were isolated from fresh NP tissue as described in the Methods and Materials section, followed by 10 ng/ml IL-1β stimulation. 12h later, mRNA was collected, and real time PCR was performed. As indicated in Figure 1C and Supplementary Figure 1B, RNA level of ghrelin was reduced by IL-1β. Moreover, NP cells were collected 72h later and examined by Western blot. As shown in Figure 1D, 1E and Supplementary Figure 1C, IL-1β reduced the expression of ghrelin in NP cells.

### Ghrelin suppresses IL-1β induced disorganized metabolism, proliferation and apoptosis in NP cells

**Figure 2:**
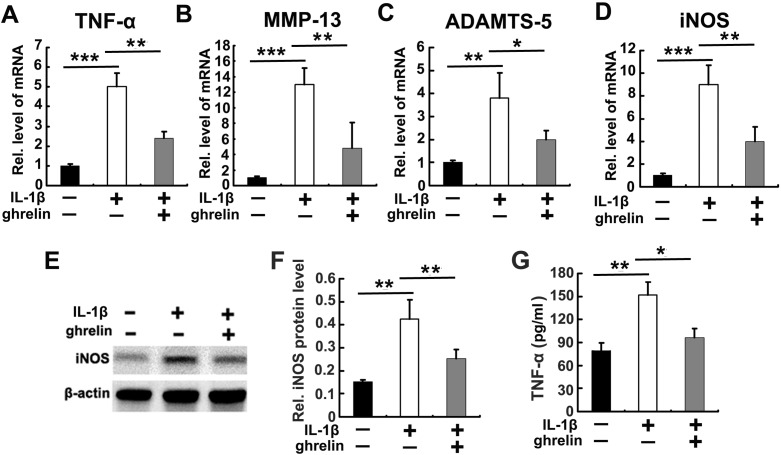
Ghrelin antagonizes IL-1β induced catabolism in NP cells **(A-D)** Ghrelin antagonizes expression of TNF-α, MMP13, ADAMTS-5 and iNOS, as detected by real time PCR. RNA extracts were collected from cultured NP cells treated with 10 ng/ml IL-1β for 12 h, with or without additional use of 50 nM ghrelin. The levels of mentioned molecules were measured using real-time PCR. **(E, F)** Ghrelin suppresses IL-1β induction of iNOS, as assayed by Western blot. NP cells were cultured in conditional medium for 72h in stimulation of 10 ng/ml IL-1β with or without treatment of 50 nM ghrelin. Consequently, iNOS was measured using Western blot. **(G)** Ghrelin inhibits expression of TNF-α in stimulation of IL-1β, as assayed by ELISA. NP cells were cultured in conditional medium for 72h in stimulation of 10 ng/ml IL-1β with or without treatment of 50 nM ghrelin, then each group was collected and TNF-α level was measured through ELISA. The values are the mean±SD. ^*^p < 0.05, ^**^ p < 0.01 and ^***^ p < 0.005 vs. Control group. Each experiment was run in triplicate.

It is known that IL-1β plays a vital role in IVD degeneration through induction of catabolic molecules [7–9]. Also, ghrelin inhibits IL-1β expression as well as its function [27]. Based on these data, we further investigated whether ghrelin antagonizes IL-1β mediated catabolism as well as inflammation in NP cells. Briefly, primary NP cells were isolated and cultured with 10 ng/ml IL-1β for 12h, in presence or absence of 50 nM ghrelin. Thereafter, cell mRNA was collected from each group and real time PCR was performed for well-established inflammation and catabolic molecules, including TNF-α, MMP-13, ADAMTS-5, and iNOS. As shown in Figure 2A–2D, IL-1β significantly increased the levels of these molecules, which was largely abolished by additional treatment of ghrelin. Furthermore, NP cells were cultured with 10 ng/ml IL-1β, in presence or absence of 50 nM ghrelin for 72h. Expression level of iNOS has been reported to be closely associated with IVD degeneration [28, 29]. In this study, iNOS were measured by western blot. As shown in Figure 2E, 2F, western blot data indicated that ghrelin suppressed IL-1β induction of iNOS in NP cells. Moreover, TNF-α is a dominant inflammatory cytokine which induces cascade of inflammation process [30, 31]. Also, TNF-α has been extensively studied as a potential therapeutic target in IVD degeneration [32, 33]. In the current study, conditional medium was collected from cultured NP cells, and TNF-α expression was analyzed using ELISA. Concisely, Figure 2G revealed that ghrelin inhibited the secretion of TNF-α mediated by IL-1β in NP cells.

Impaired proliferation and exaggerated apoptosis of NP cells are observed in IVD degeneration. In this study, IL-1β treatment decreased the proliferation and enhanced apoptosis of NP cells, while additional administration of ghrelin repressed the effect of IL-1β in cell proliferation and apoptosis, as measured by EdU (Supplementary Figure 2A) and Western blot of Caspase-3 cleavage (Supplementary Figure 2B).

### Ghrelin antagonizes IL-1β mediated NF-κB signaling pathway in NP cells

**Figure 3:**
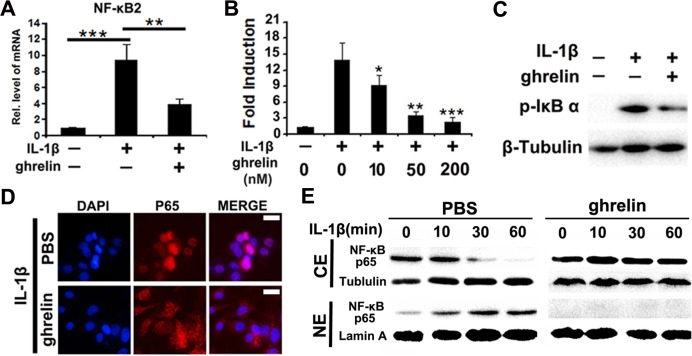
Ghrelin represses IL-1β mediated activation of NF-κB signaling pathway **(A)** IL-1β enhances NF-κB2 expression level, which is largely abolished by additional treatment of ghrelin, as detected by real time PCR. NP cells were cultured with stimulation of 10 ng/ml IL-1β, in presence or absence of 50 nM ghrelin for 1h. The mRNA was then collected and real time PCR was performed. **(B)** Ghrelin suppresses IL-1β induction of NF-κB signaling pathway in a dose dependent manner, as assayed by reporter gene assay. NP cells were cultured with stimulation of 10 ng/ml IL-1β, in presence of various doses of ghrelin for 24h, followed by reporter gene assay. **(C)** Ghrelin antagonizes IL-1β induced phosphorylation of IκB-α, detected by Western blot. NP cells were cultured with stimulation of 10 ng/ml IL-1β, in presence or absence of 50 nM ghrelin for 24h. pIκB-α levels were analyzed by Western blot. **(D)** Ghrelin inhibits nuclear transaction of NF-κB p65 in stimulation of IL-1β, as assayed by cell immunostaining. NP cells were cultured with stimulation of 10 ng/ml IL-1β, in presence or absence of 50 nM ghrelin for 1h, and cell immunostaining was performed. (**E**) Nuclear transaction of NF-κB P65 was analyzed by Western blotting. Western blot of NF-κB P65 was performed using cytoplasmic (CE) and nuclear (NE) extracts of IL-1β-treated NP cells in the presence and absence of 50 nM ghrelin. Tubulin and lamin A served as cytoplasmic and nuclear controls, respectively. The values are the mean±SD. ^*^p < 0.05, ^**^ p < 0.01 and ^***^ p < 0.005 vs. Control group. Each experiment was repeated for three times.

IL-1β mediated NF-κB signaling pathway is critical for IVD degeneration. To investigate whether ghrelin represses IL-1β mediated activation of NF-κB signaling in NP cells, NP cells were cultured with 10 ng/ml IL-1β for 1h, in presence or absence of indicated dose of ghrelin. As indicated in Figure 3A, NF-κB2, a key part of NF-κB signaling pathway, increased after administration of IL-1β, while this function of IL-1β was inhibited by additional treatment of 50 nM ghrelin. Furthermore, reporter gene assay was performed for NF-κB signaling, and Figure 3B indicates that ghrelin suppressed IL-1β induction of the NF-κB signaling pathway in a dose-dependent manner.

Phosphorylation of IκB is a critical feature for activation of NF-κB signaling pathway. In the present study, total protein extract was collected from each group, and pIκB-α was analyzed by western blot. Figure 3C revealed that IL-1β enhanced phosphorylation of IκB-α, which was inhibited by additional treatment of ghrelin. To further test the role of ghrelin in IL-1β mediated NF-κB signaling activation, nuclear transaction of NF-κB p65 was assessed in NP cells. Immunostaining (Figure 3D) as well as Western blot (Figure 3E) results showed that ghrelin reduced nuclear distribution of p65 in stimulation with IL-1β.

### Ghrelin improves the impaired anabolism of NP tissue in a rabbit IVD degeneration model

**Figure 4:**
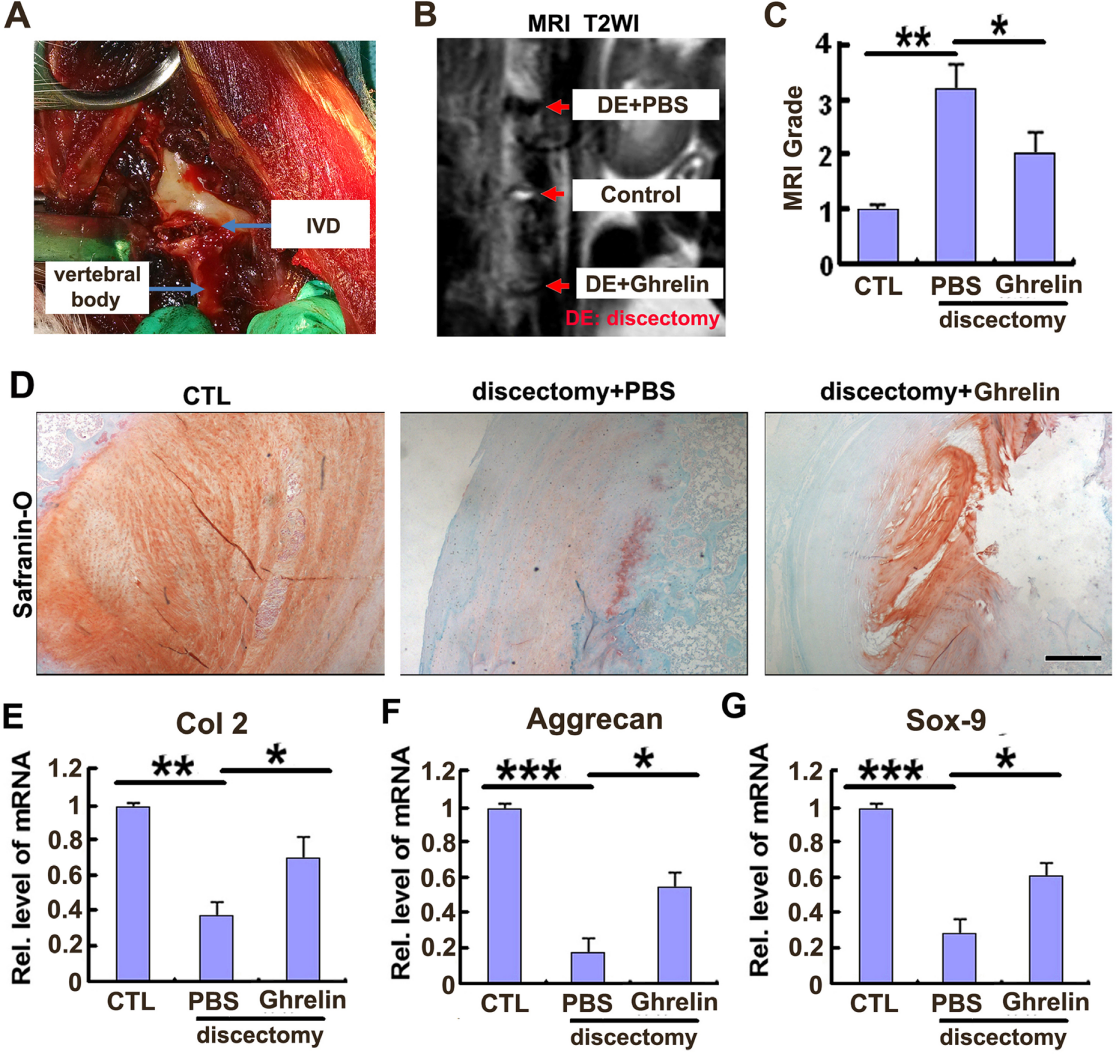
Ghrelin protects against destruction of NP tissue in a rabbit IVD degeneration model **(A)** The representative image during surgery. **(B)** Signal of IVD destruction was displayed in W2 weighted image in the rabbit model, while ghrelin treatment markedly improved the IVD structure, as assayed by MRI. 8 weeks after surgery, rabbits were anesthetized and MRI was performed in each group (N=7 per group). **(C)** Ghrelin reduced the MRI grade in IVD discectomy model, as indicated by MRI grading system. **(D)** Ghrelin treatment maintained extracellular matrix in NP tissue. IVD samples were isolated from each experimental group, and Safranin-O staining was performed. (**E-G)** Ghrelin treatment attenuated the reduction of Col 2, aggrecan as well as Sox-9 in NP tissue, as assayed by real time PCR. NP tissue was collected and real time PCR was performed. The values are the mean±SD. ^*^p < 0.05, ^**^ p < 0.01 and ^***^ p < 0.005 vs. Control group. Each experiment was repeated for 3 times. Scale bar=250μm.

One feature of IVD degeneration is metabolism imbalance, which implies increased catabolism and reduced anabolism. It has been demonstrated that ghrelin was closely associated with tissue regeneration in several different conditions [34], together with the data of the present study that ghrelin antagonizes expression of degeneration associated molecules *in vitro*, prompted us to investigate the role of ghrelin in a well-accepted rabbit IVD degeneration model [23]. Figure 4A indicates the representative image during surgery, and a partial discectomy (3mmx3mmx4mm) procedure performed on the L3/4, L4/5 or L5/6 levels randomly as described in the Materials and Methods section. Thereafter, HA scaffold was implanted into the NP defect in 2 IVD levels, in the absence or presence of 100 ng recombinant ghrelin. The left IVD level was used as negative control. 8 weeks after surgery, rabbits were analyzed using MRI; Figure 4B indicates that ghrelin improved the NP signal in the rabbit model. MRI grade, a well-established scoring system for severity of degeneration in IVD, was assessed through Pfirrmann's classification scores as previously reported [26]. Figure 4C reveals that ghrelin significantly attenuated the MRI grade in this IVD discectomy degeneration model. Subsequently, IVD samples were isolated and Safranin-O staining was performed. Figure 4D shows that ghrelin treatment maintained extracellular matrix in NP tissue. Moreover, NP tissue was collected from each group, and real time PCR was performed. As shown in Figure 4E–4G, ghrelin treatment attenuated the reduction of Col 2, aggrecan as well as Sox-9. To further testify whether local administration of ghrelin alleviated degeneration of NP tissue *in vivo*, NP tissues were isolated from this model, and levels of degeneration biomarkers in rabbit NP tissue, including MMP-3, ADAMTS-4 and ADAMTS-5 were examined using real time PCR [9, 35]. As shown in Supplementary Figure 3A-3C, the mentioned molecules were enhanced in degenerative IVD tissue, while ghrelin attenuated the election of the mentioned degenerative molecules in this rabbit IVD degeneration model.

### Ghrelin promotes anabolism of NP cells through activation of Akt signaling pathway

**Figure 5:**
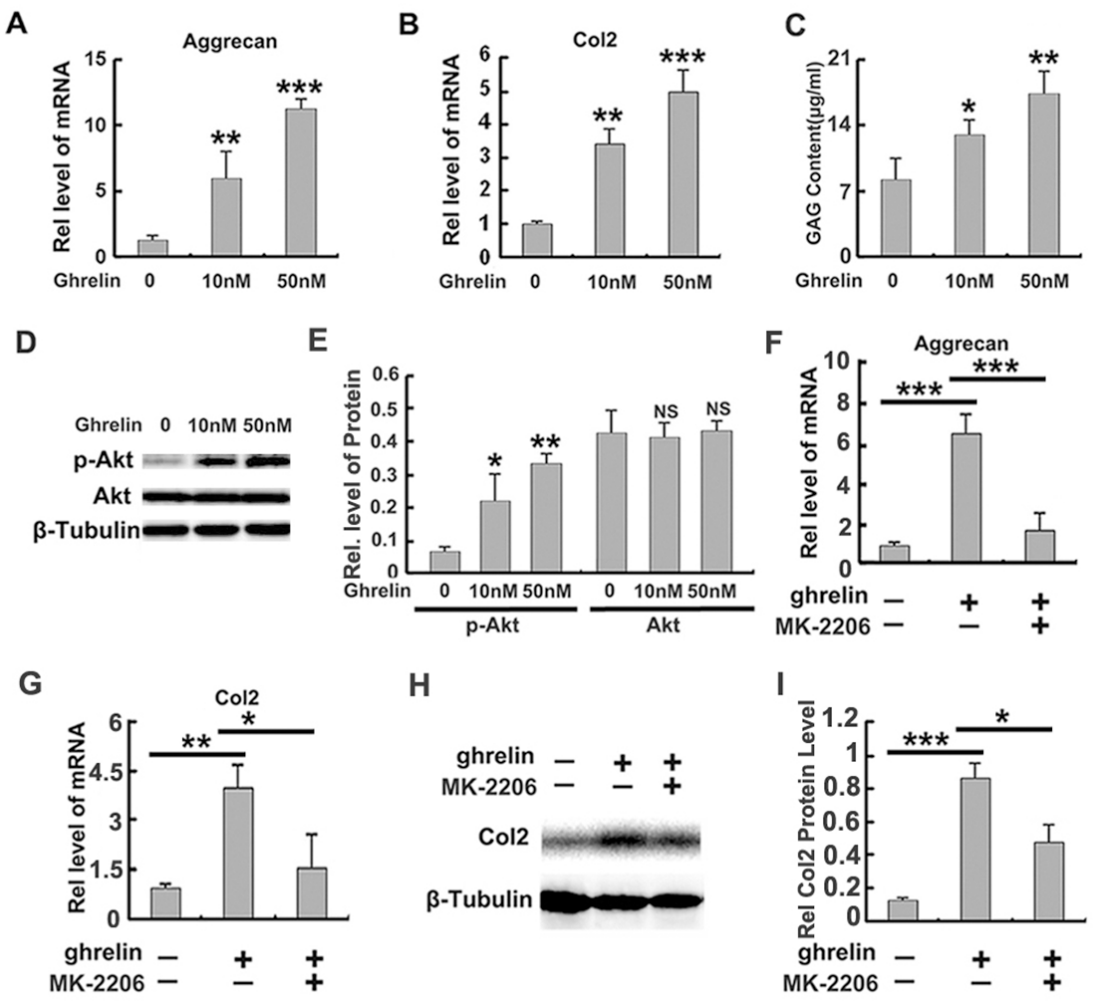
Ghrelin enhances anabolism of NP cells through Akt signaling pathway **(A, B)** Ghrelin elevates production of aggrecan and Col2 in a dose dependent manner, as measured by real time PCR. Primary human NP cells were isolated and cultured in presence of various doses of ghrelin for 12h, followed by real time PCR assay. **(C)** Ghrelin enhances synthesis of GAG extracellular matrix, as measured by GAAB assay. Conditional medium was collected from each group after culturing NP cells for 72h, and GAAB assay was performed. **(D, E)** Ghrelin enhances phosphorylation of Akt, as assayed by Western blot. **(F, G)** Anabolic ability of ghrelin is suppressed by MK-2206, a commonly used Akt signaling pathway. Total mRNA was collected from each indicated group and real time PCR was performed. **(H, I)** Col 2 expression is elevated by ghrelin, while MK-2206 antagonizes this function of ghrelin, as detected by Western blot. The NP cells were cultured for 72h and Western blot was performed. The values are the mean±SD. ^*^p < 0.05, ^**^ p < 0.01 and ^***^ p < 0.005 vs. Control group. Each experiment was repeated for 3 times.

Dampened anabolism is a critical feature in IVD degeneration. Several studies have reported that improvement of anabolism was beneficial for homeostasis of NP structure [5]. It has also been shown that aggrecan and Col 2 were key matrix components in NP tissue. In this study, primary NP cells were cultured with PBS or indicated doses (0, 10 nM, 50 nM) of ghrelin for 12h. Thereafter, mRNA was collected, and levels of aggrecan and Col 2 were measured by time PCR. As shown in Figure 5A, 5B, aggrecan and Col 2 mRNA levels were significantly elevated by ghrelin in a dose dependent manner. Moreover, NP cells were cultured for 72h, and conditional medium was collected from each group, followed by GAAB assay. Figure 5C revealed that ghrelin mediated synthesis of GAG extracellular matrix in a dose dependent manner.

It is reported that ghrelin activates Akt signaling pathway in several conditions [36]. In addition, Akt signaling pathway has been shown to be closely associated with anabolism of NP cell [20]. Based on these reports, we investigated whether the function of ghrelin in anabolism of NP cell relies on Akt signaling pathway. Primary human NP cells were cultured in presence of 0, 10 nM or 50 nM ghrelin. Figure 5D and 5E indicate that ghrelin induced phosphorylation of Akt in NP cells in a dose dependent manner, which implies activation of Akt signaling pathway. To further study the requirement of Akt signaling in ghrelin mediated anabolism, primary NP cells were cultured with PBS or 50 nM ghrelin in presence or absence of MK-2206, a commercial inhibitor of Akt signaling pathway, for 12h. Thereafter, mRNA was collected, and real time PCR indicated that ghrelin induced aggrecan (Figure 5F) and Col2 (Figure 5G) production was suppressed by additional use of MK-2206. Moreover, NP cell was cultured for 72h, and Western blot of Col 2 (Figure 5H, 5I) revealed that MK-2206 remarkably antagonized the function of ghrelin in anabolism. Collectively, this set of experiments implies that the anabolic role of ghrelin at least partially depends on the activation of Akt signaling pathway.

### GHSR is required for the protective role of ghrelin in anabolism of NP cells

**Figure 6:**
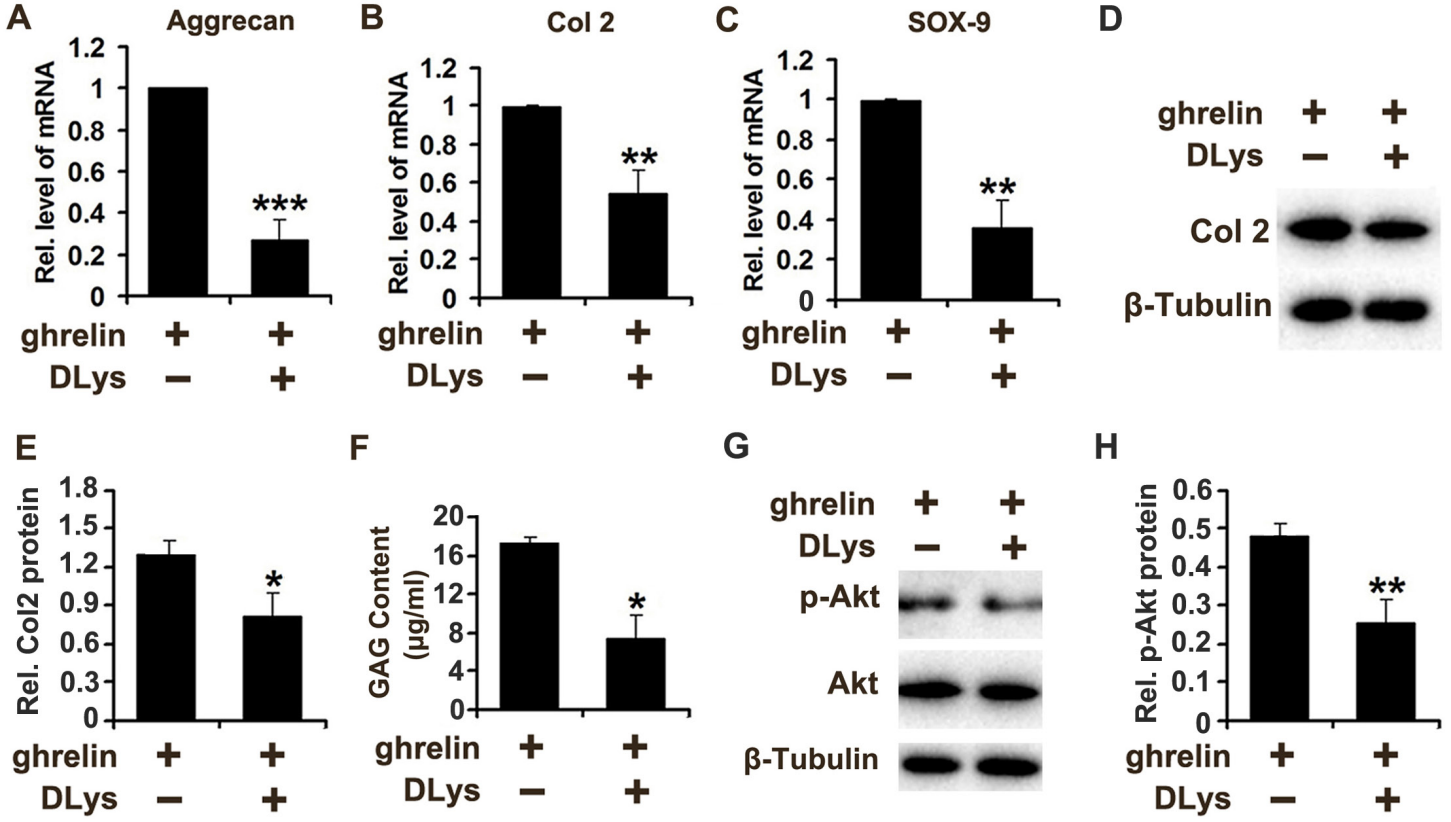
Ghrelin enhances anabolism of NP cells through interacting with GHSR **(A-C)** Inhibition of GHSR suppressed the levels of Aggrecan, Col 2 and Sox-9 induced by ghrelin, as assayed by real time PCR. NP cells were cultured for 12h and total mRNA was collected, followed by real time PCR. **(D, E)** Ghrelin mediated Col 2 synthesis was reduced by antagonization of GHSR through DLys, as measured by Western blot. NP cells were cultured for 72h and protein was collected, followed by Western blot. **(F)** DLys abolished ghrelin mediated synthesis of GAG extracellular matrix. Conditional medium was collected from each group after culture of NP cells for 72h, and GAAB assay was performed. **(G, H)** Ghrelin mediated activation of Akt signaling pathway was diminished by DLys, as assessed by Western blot. NP cells were cultured with 50 nM ghrelin in presence or absence of DLys for 12h and protein was collected, followed by Western blot. The values are the mean±SD. ^*^p < 0.05, ^**^ p < 0.01 and ^***^ p < 0.005 vs. Control group. Each experiment was repeated for 3 times.

Growth hormone secretagogue receptor (GHSR) is required for the function of ghrelin in various conditions [37, 38]. Also, ghrelin enhances anabolism of NP cells (Figure 5). Based on these findings, we further demonstrated the potential involvement of ghrelin/GHSR pathway in anabolism of NP cells. Human primary NP cells were stimulated with ghrelin in absence or presence of [D-Lys(3)]-GHRP-6 (DLys), a commonly used inhibitor for GHSR. NP cells were cultured for 12h, followed by real time PCR for aggrecan, Col 2 and Sox-9. As shown in Figure 6A–6C, inhibition of GHSR suppressed ghrelin induction of these biomarkers of anabolism. Furthermore, NP cells were cultured for 72h as mentioned above; and Western blot and GAG synthesis were assessed. Figure 6D and 6E indicate that ghrelin mediated Col 2 synthesis was reduced by DLys. Moreover, conditional medium was collected from each group, and GAAB assay was performed. Figure 6F reveales that DLys significantly abolished ghrelin mediated synthesis of GAG extracellular matrix. Additionally, ghrelin induced activation of Akt signaling pathway was diminished by DLys (Figure 6G, 6H).

## DISCUSSION

IVD degeneration, which has been extensively investigated for years, is a main cause of low back pain [39]. Nevertheless, to date, its molecular mechanism remains largely unclear. Previous studies have suggested that NP tissue degeneration is essential for protrusion of IVD, thus various studies have focused on how to maintain homeostasis of NP structure [40]. In the current study, we detected expression of ghrelin in both human NP tissue and isolated primary NP cell, which implied that ghrelin might be associated with degeneration of IVD. One critical feature of IVD degeneration is enhanced catabolism of NP cell [40]. Among all the cytokines, IL-1β has been well accepted as a critical inducer of degeneration of IVD tissue [18], and has provided a potential target for treatment of IVD degeneration. In NP cells, ghrelin level was reduced following the stimulation of IL-1β, which suggests potential interaction between NP cells homeostasis and ghrelin. The enhanced expression of degeneration related molecules, including ADAMTS-5, MMP13, iNOS and TNF-α in NP cells are commonly observed in IVD degeneration [9, 41–43]. In the current study, the degeneration and inflammatory reaction of degenerative NP cells was dramatically suppressed by exogenous ghrelin, which implies the promising effect of ghrelin in repressing NP tissue catabolism. Furthermore, reduced proliferation and increased NP cell apoptosis have been widely studied in IVD degeneration process [44, 45]. In this study, ghrelin treatment reversed the disorganized proliferation and apoptosis activity of IL-1β stimulated NP cells. Moreover, a rabbit IVD degeneration model was established [23], and ghrelin administration alleviated enhanced levels of degeneration associated biomarkers, including ADAMTS-4, ADAMTS-5 and MMP-3 in NP tissue [9, 35].

The NF-κB pathway is critical for mediating IL-1β activity [46]. Moreover, NF-κB signaling pathway has been identified as a key mediator of age-dependent disc degeneration [47]. Different studies have shown that stimulation of NF-κB signaling could accelerate, while inhibition of this signaling could attenuate, disc degenerative diseases associated with aging [48, 49]. Phosphorylation of IκB-α is a commonly tested parameter for the activation of NF-κB signaling pathway [50]. In our study, we found that the level of pIκB-α was dramatically increased in cells treated with IL-1β, and this was antagonized by exogenous ghrelin. Furthermore, in the present study, nuclear transaction of p65, a well-known parameter for activation of NF-κB signaling pathway [51], was greatly inhibited by ghrelin administration. All these data indicated that ghrelin suppresses the activity of NF-κB signaling pathway, which is in line with previous reports [20, 21]. Collectively, these data suggested that the excessive activation of the NF-κB signaling pathway in the presence of IL-1β was inhibited by ghrelin, which might therefore attenuate NP degeneration process.

Re-establishing extracellular matrix of degenerative NP is a critical direction for treatment of IVD degeneration [52]. Among all extracellular matrix components, aggrecan and col 2 are the main parts in NP tissue, and are the key parameters for anabolic ability of NP cell [53]. In this study, primary NP cells from patients with DDD were cultured with or without ghrelin, and revealed that ghrelin enhanced production of aggrecan and Col 2. Extracellular matrix destruction in NP tissue is a critical feature of IVD degeneration [54], and rabbit IVD degeneration models were used for investigation of DDD treatment [23, 55]. In the present study, we established a rabbit IVD degeneration model, which indicated that ghrelin maintained the extracellular matrix in NP tissue. Ghrelin has been shown to play a protective role in homeostasis of several tissues. In the present study, ghrelin was administrated in combination with hyaluronic acid scaffold, which maintained structure of IVD in the rabbit model. According to obtained data, local delivery of ghrelin might directly stimulate anabolism and antagonize the degrading procedure mediated by metalloproteinases in the NP tissue residue. On the other hand, elevation of local ghrelin level might fight against the inflammation reaction in the degenerative IVD tissue, which is a potential therapeutic target for treatment of IVD degeneration [56, 57]. These two factors may contribute to the protective role of ghrelin in this rabbit model.

Akt signaling pathway is closely associated with anabolic ability of NP cells [36], and ghrelin is reported to activates Akt signaling pathway [20]. Herein, primary NP cells were cultured, and ghrelin promoted Akt phosphorylation, indicating that ghrelin activates Akt signaling pathway in NP cells. To further determine whether Akt signaling pathway is required for the anabolic function of ghrelin in NP cells; MK-2206, a commonly used antagonist of Akt signaling pathway [58], was used and suppressed ghrelin mediated anabolism in NP cells. This suggested that in promotion of anabolism in NP cells, gherlin at least partially relied on Akt signaling pathway.

GHSR is the ghrelin receptor indispensable for the protective role of ghrelin in various diseases [59, 60], while DLys is a common inhibitor of GHSR to investigate the ghrelin functions [61, 62]. In the current study, following the use of DLys in NP cells, the ghrelin induction of anabolism was largely repressed, which implies the interaction between ghrelin and GHSR in induction of extracellular matrix in NP cells. In conclusion, ghrelin seems to have protective function against degeneration of IVD by maintaining the homeostasis of NP cells, which provides a potential therapeutic target for DDD.

## MATERIALS AND METHODS

### Ethics statement

Specimens were collected from 34 patients who underwent lumbar operations between November 2013 and June 2016 in Qilu Hospital of Shandong University, Jinan, China. The present study was approved by the Medical Ethical Committee of Shandong University Qilu Hospital. Informed consent documents were obtained from all patients involved in this research.

### Isolation and culture of primary IVD cells

Human lumbar IVD samples were obtained from patients undergoing posterior lumbar interbody fusion (PLIF) operations or lumbar discectomy surgeries for degenerative diseases (n = 34 patients, aged 23–48). Primary IVD cells were isolated and cultured according the previously reported method [22]. All of the samples were anonymized, and only patients’ genders and ages were recorded. Disc tissues were transferred to the laboratory immediately post- dissection/surgery. Briefly, discs were first washed with cold, aseptic phosphate-buffered saline (PBS) to remove residual blood. IVD tissues were then carefully separated and cut into fragments of ∼ 1 mm^3^. Consequently, tissue samples were separately digested with trypsin and type II collagenase (Sigma-Aldrich, Ltd, China), following the filtration of NP cells through a 200-mesh sieve. The isolated NP cells were seeded as a monolayer and cultured in DMEM/F12 media (Hyclone, Thermo Co., USA) containing 15 % FCS and 1 % PS under standard incubation conditions (37°C, 95 % air, 5 % CO2, pH 7.2) for approximately 3 weeks. After the primary cells adhered to the bottom of the culture bottle, the culture media was replaced every 3 days, and the indicated experiments followed.

### Surgical technique

All animal studies were done in compliance with the regulations and guidelines of Shandong University Institutional Animal Care and conducted according to the IACUC guidelines. The rabbits were tranquillized by intramuscular injection of xylazine (3 mg/kg) and ketamine (40 mg/kg) and were then anaesthetized with sodium pentobarbital (30 mg/kg, Pharma Inc, Nanjing; PRC). All surgical procedures took place under aseptic conditions. The rabbits were placed on the operating table in a prone position. An anterolateral retroperitoneal approach was used to expose three consecutive levels of the rabbit IVD, comprising L3/4, L4/5 and L5/6. Experimental intervertebral discs were injured in the anterolateral AF as previously reported [23], and partial discectomy was performed in NP tissue (Figure 4A). To exclude a spinal level bias, discectomy and control groups were randomly allocated to three disc levels. The experimental discs were assigned to three groups (n=7 per group): (1) Control group: the IVD level was exposed but without any injury of AF or NP; (2) Discectomy+PBS group: discectomy was performed in NP and hyaluronic acid scaffold with PBS was implanted in the site of NP defect; (3) Discectomy+ghrelin group: discectomy was performed in NP and hyaluronic acid scaffold with 200 ng recombinant ghrelin (sc-364689) was implanted in the site of NP defect;. Following surgery, the rabbits were permitted free cage activity and food and water ad libitum. In addition, no surgery-related complications or neurological symptoms were observed.

### Immunofluorescence staining

Immunofluorescence was used to investigate the expression pattern of ghrelin in NP cells. Briefly, primary human NP cells were cultured on coverslips, followed by immunofluorescence staining (antibody catalogue number: sc50297 and ab57222) according the previously reported method [24]. Moreover, NP cells were stimulated with 10 ng/ml IL-1β in the presence or absence of ghrelin for 1 hour. Consequently, immunofluorescence staining of NF-κB p65 (antibody catalogue number: ab97726) was performed on these cells as described previously [24] and examined using a confocal fluorescence microscope system.

### Histology and immunohistochemistry

IVD tissues from each animal were collected, fixed in 4% PFA for 3 days and decalcified for 2 weeks in 10% w/v EDTA before dehydrated, and consequently embedded in paraffin and cut in 5μm thick sections. Serial sections were stained with Safranin-O/fast green/iron hematoxylin. Dissected human NP tissues were sectioned, deparaffinized, rehydrated, and incubated in Tris buffer (10 Mm Tris-HCl (pH 8.0), 150mM NaCl). The samples were then incubated with rabbit anti-mouse ghrelin antibody (sc50297, 1:100) at 4°C overnight. The sections were then incubated for 30 min with biotinylated anti-rabbit IgG (Vector, Burlingame, CA) and subsequently stained using a biotin-streptavidin-peroxidase protocol (Vector). Horseradish peroxidase (HRP) activity was detected using 3, 3′-diaminobenzidine and H_2_O_2_. Slides were counterstained with 0.5% Methyl green.

### Western blot

Primary human NP cells from each indicated group were homogenized and proteins were collected. Proteins were resolved on a 10% SDS-polyacrylamide gel and electroblotted on a nitrocellulose membrane. After blocking in 5% nonfat dry milk in Tris buffer-saline-Tween 20 (10mM Tris-HCl, pH 8.0; 150mM NaCl; and 0.5% Tween 20), blots were incubated with polyclonal anti-ghrelin (sc50297, diluted 1:1000), monoclonal-anti-ghrelin (ab57222, diluted 1:1000), anti-collagen 2 (sc-52658, diluted 1:500), anti-phosphorylated IκB-α (pIκB-α, sc-7977, diluted 1:1000), anti-iNOS (sc-7271, diluted 1:1000), anti- NF-κB p65 (ab97726, diluted 1:1000) or anti-Caspase-3 (ab32351, diluted 1:500) antibody for 1 h. After washing, the secondary antibody (horseradish peroxidase conjugated anti-rabbit immunoglobulin; 1:2000 dilution) was added, and bound antibody was visualized using an enhanced chemiluminescence system (Amersham Life Science, Arlington Heights, IL, USA).

### Real-time PCR

Total mRNA was extracted from primary NP cells of each indicated group using RNeasy kit (Qiagen), and first-strand cDNA was generated with ImProm-II reverse transcription system (Promega). Real-time PCR was performed with SYBR Green I dye. Data from each sample were normalized to GAPDH. Primers used for real-time RT-PCR were designed to generate products between 100bp and 200bp in length. Total RNA was isolated from NP cells. Then they were reverse-transcribed to cDNA. Real-time PCR was performed with the sequence-specific primers as shown in Tables 1 and 2. The presence of a single specific PCR product was verified for each gene by melting curve analysis. The experiments were run in triplicate.

**Table 1:** Sequences of primers used for quantitative real-time PCR

	Gene	Primer sequence (5′-3′)	Annealing temperature (°C)
Human	Ghrelin	Forward: TGAGCCCTGAACACCAGAGAG	60
	(Primer 1)	Reverse: AAAGCCAGATGAGCGCTTCTA	
	ghrelin	Forward: ATGCTCTGGCTGGACTTGG	60
	(Primer 2)	Reverse: CTGGTGGCTTCTTCGACTCC	
	Aggrecan	Forward: AATGCTGGTACTCCAAACCC	62
		Reverse: CTGGATCGTTATCCAGCAAACAGC	
	Col 2	Forward: ACTAGTCATCCAGCAAACAGCCAGG	62
		Reverse: TTGGCTTTGGGAAGAGAC	
	ADAMTS-5	Forward: GCAGTATGACAAGTGCGGAGT	62
		Reverse: CAGGGCTAAATAGGCAGTGAA	
	MMP-13	Forward: ACTTTGTTGCCAATTCCAGG	62
		Reverse: TTTGAGAACACGGGGAAGAC	
	iNOS	Forward: ACAGGAGGGGTTAAAGCTGC	60
		Reverse: TTGTCTCCAAGGGACCAGG	
	NF-κB2	Forward: CAGTGAGAAGGGCCGAAAGAC	65
		Reverse: CAGGGGCAGGGAGAAGGAG	
	Sox-9	Forward: ATGAAGATGACCGACGAGCA	60
		Reverse: CAGTCGTAGCCTTTGAGCAC	
	GAPDH	Forward: AGAAGGCTGGGGCTCATTTG	
		Reverse: AGGGGCCATCCACAGTCTTC	60

**Table 2:** Sequences of primers used for quantitative real-time PCR

	Gene	Primer sequence (5′-3′)	Annealing temperature (°C)
Rabbit	Col 2	Forward: GTGGTGACAAAGGCGAAAAG	62
		Reverse: CCTTCTCGTCAAATCCTCCA	
	Aggrecan	Forward: GGAGTTCTTTTTGGGAGTGGT	62
		Reverse: CAGGTCAGGGATTCTGTGTGT	
	SOX-9	Forward, CGAACGCACATCAAGACG	62
		Reverse: AAGGTGGAGTAGAGGCTGGA	
	ADAMTS-4	Forward:GACCTTCCGTGAAGAGCAGTGT	62
		Reverse: CCTGGCAGGTGAGTTTGCAT	
	ADAMTS-5	Forward: CCTGGCAGGTGAGTTTGCAT	62
		Reverse:GGAGAACATATGGTCCCAACGT	
	MMP-3	Forward: GCCAAGAGATGCTGTTGATG	62
		Reverse: AGGTCTGTGAAGGCGTTGTA	
	GAPDH	Forward:CTCTGGCAAAGTGATG	60
		Reverse: TCCTGGAAGATGGTGATG	

### Reporter gene assay

To examine whether ghrelin inhibited IL-1β-mediated transactivation of NF-κB-dependent reporter genes, reporter gene assay was performed according the previously described approach, with slight modifications [25]. Briefly, after reaching ∼50% confluence, NP cells were transfected with 1μg of the p6XNF-κB-Luc reporter plasmid and 1μg of the pSVGal plasmid (internal control), using Lipofectamine 2000 reagent (Invitrogen). 48 hours post- transfection, the cells were starved overnight and then stimulated for additional 6h with 10 ng/ml IL-1β, in the presence or absence of various doses of ghrelin. Finally, the luciferase activity was detected with a fluorescence microscope (Leica, Weitsbaden, Germany). Magnetic resonance imaging analysis.

Magnetic resonance images (MRI) and lateral plain radiographs were performed under general anesthesia (sodium pentobarbital, 30 mg/kg) on the 8^th^ week post- surgery (*n* = 7). MRI examinations were performed using a 1.5-T Imager with a quadrature extremity coil receiver. Mid-sagittal T2-weighted images were obtained in the following settings: fast spin echo sequence with time to repetition (TR) of 3500 ms, time to echo (TE) of 100 ms, 320(h) × 256(v) matrix; field of view 260; and number of excitations 4; and slice thickness 2 mm with a 0-mm gap. The MRI scans were evaluated by 2 blinded observers using the Pfirrmann's classification scores [26] based on changes in the degree and area of signal intensity: 1 = normal, 2 = Inhomogeneous structure, high signal intensity, 3 = moderate decrease in signal intensity, but slightly narrowing the disc height, 4 = severe decrease in signal intensity, moderately narrowing the disc height.

### DMMB assay of GAG content in NP cells

Conditioned medium was collected from each treatment group of cultured human primary NP cells, and the content of GAG released from cells was quantified using DMMB dye (Polysciences, Warrington, PA, USA). Culture medium was pre-treated with 0.5units/ml of hyaluronidase (Seikagaku, Tokyo, Japan) at 37°C for 3h in order to remove exogenous HA which might interfere with the DMMB assay. Digests (in duplicate) were mixed with DMMB in 96-well plates and red at 520 nm using SpectraMax 384 Microplate Reader (Molecular Devices, Sunnyvale, CA, USA). The amount of GAG in the conditioned medium was extrapolated using chondroitin-6-sulfate sodium salt from Shark cartilage (Sigma e Aldrich, St. Louis, MO, USA) as a standard. The data were shown as the mean of GAG released into the condition media from three sets of experiments (treated in separate wells).

### ELISA assays for TNF-α

Primary NP cells were cultured with 10 ng/ml IL-1β in presence or absence of 50 nM ghrelin for 72h. ELISA was used to assay the level of TNF-α according the previously reported approach [25]. TNF-α level was examined using a commercial kit (eBioscience), according to manufacturer instructions.

### EdU proliferation assay

The NP cells were seeded at the proper density in 96-well plates with different treatments for 72 h. The Cell-Light™ EdU DNA Cell Proliferation Kit (Ribobio, Guangdong, China) was used to stain and detect proliferation of the cells according to the manufacturer instructions. Cells were analyzed with a fluorescence microscope (Leica, Weitsbaden, Germany) using Cy5 (proliferative cells) and Hoechst33342 (all cells) filter.

### Statistical analysis

For comparison of treatment groups, we performed unpaired t-tests (Mann-Whitney), paired t-tests, and one-way or two-way ANOVA (where appropriate). For ANOVA, we used Bonferroni *post hoc* analysis to compare treatment groups. All statistical analyses were performed using GraphPad Prism Software (version 4.01). P < 0.05 was considered significantly different.

## SUPPLEMENTARY MATERIALS FIGURES AND TABLES



## Abbreviations

ADMATS-4: a disintegrin and metalloproteinase with thrombospondin motif-4
ADMATS-5: a disintegrin and metalloproteinase with thrombospondin motif-5
TNF-α: tumor necrosis factor α
IL-1β: interleukin-1β
Col 2: collagen 2
NF-κB: nuclear factor-kappaB
pIκB-α: phosphorylated IκB-α
iNOS: inducible nitric oxide synthase
MMP-3: matrix metalloproteinase-3
MMP-13: matrix metalloproteinase-13.

## References

[1] Xu J, E XQ, Wang NX, Wang MN, Xie HX, Cao YH, Sun LH, Tian J, Chen HJ, Yan JL (2016). BMP7 enhances the effect of BMSCs on extracellular matrix remodeling in a rabbit model of intervertebral disc degeneration. FEBS J.

[2] Zhao YP, Tian QY, Liu B, Cuellar J, Richbourgh B, Jia TH, Liu CJ (2015). Progranulin knockout accelerates intervertebral disc degeneration in aging mice. Sci Rep.

[3] Hwang PY, Jing L, Chen J, Lim FL, Tang R, Choi H, Cheung KM, Risbud MV, Gersbach CA, Guilak F, Leung VY, Setton LA (2016). N-cadherin is key to expression of the nucleus pulposus cell phenotype under selective substrate culture conditions. Sci Rep.

[4] Yang H, Yuan C, Wu C, Qian J, Shi Q, Li X, Zhu X, Zou J (2016). The role of TGF-beta1/Smad2/3 pathway in platelet-rich plasma in retarding intervertebral disc degeneration. J Cell Mol Med.

[5] Liu H, Pan H, Yang H, Wang J, Zhang K, Li X, Wang H, Ding W, Li B, Zheng Z (2015). LIM mineralization protein-1 suppresses TNF-alpha induced intervertebral disc degeneration by maintaining nucleus pulposus extracellular matrix production and inhibiting matrix metalloproteinases expression. J Orthop Res.

[6] Daniels J, Binch AA, Le Maitre CL (2017). Inhibiting IL-1 signalling pathways to inhibit catabolic processes in disc degeneration. J Orthop Res.

[7] Jin H, Shen J, Wang B, Wang M, Shu B, Chen D (2011). TGF-beta signaling plays an essential role in the growth and maintenance of intervertebral disc tissue. FEBS Lett.

[8] Gu SX, Li X, Hamilton JL, Chee A, Kc R, Chen D, An HS, Kim JS, Oh CD, Ma YZ, van Wijnen AJ, Im HJ (2015). MicroRNA-146a reduces IL-1 dependent inflammatory responses in the intervertebral disc. Gene.

[9] Kim JS, Ellman MB, Yan D, An HS, Kc R, Li X, Chen D, Xiao G, Cs-Szabo G, Hoskin DW, Buechter DD, Van Wijnen AJ, Im HJ (2013). Lactoferricin mediates anti-inflammatory and anti-catabolic effects via inhibition of IL-1 and LPS activity in the intervertebral disc. J Cell Physiol.

[10] Gao Q, Horvath TL (2008). Neuronal control of energy homeostasis. FEBS Lett.

[11] Caminos JE, Gualillo O, Lago F, Otero M, Blanco M, Gallego R, Garcia-Caballero T, Goldring MB, Casanueva FF, Gomez-Reino JJ, Dieguez C (2005). The endogenous growth hormone secretagogue (ghrelin) is synthesized and secreted by chondrocytes. Endocrinology.

[12] Liu C, Huang J, Li H, Yang Z, Zeng Y, Liu J, Hao Y, Li R (2016). Ghrelin accelerates wound healing through GHS-R1a-mediated MAPK-NF-kappaB/GR signaling pathways in combined radiation and burn injury in rats. Sci Rep.

[13] Suzuki H, Masaoka T, Hosoda H, Ota T, Minegishi Y, Nomura S, Kangawa K, Ishii H (2004). Helicobacter pylori infection modifies gastric and plasma ghrelin dynamics in Mongolian gerbils. Gut.

[14] Moskalev EA, Jandaghi P, Fallah M, Manoochehri M, Botla SK, Kolychev OV, Nikitin EA, Bubnov VV, von Knebel Doeberitz M, Strobel O, Hackert T, Buchler MW, Giese N (2015). GHSR DNA hypermethylation is a common epigenetic alteration of high diagnostic value in a broad spectrum of cancers. Oncotarget.

[15] Sun N, Wang H, Wang L (2016). Ghrelin inhibits oxLDL-induced inflammation in RAW264.7 mouse macrophages through down-regulation of LOX-1 expression via NF-kappaB signaling pathway. Cell Mol Biol (Noisy-le-grand).

[16] Maduzia D, Matuszyk A, Ceranowicz D, Warzecha Z, Ceranowicz P, Fyderek K, Galazka K, Dembinski A (2015). The influence of pretreatment with ghrelin on the development of acetic-acid-induced colitis in rats. J Physiol Pharmacol.

[17] Li J, Volk A, Zhang J, Cannova J, Dai S, Hao C, Hu C, Sun J, Xu Y, Wei W, Breslin P, Nand S, Chen J (2017). Sensitizing leukemia stem cells to NF-kappaB inhibitor treatment in vivo by inactivation of both TNF and IL-1 signaling. Oncotarget.

[18] Li J, Guan H, Liu H, Zhao L, Li L, Zhang Y, Tan P, Mi B, Li F (2017). Epoxyeicosanoids prevent intervertebral disc degeneration in vitro and in vivo. Oncotarget.

[19] Yang SD, Ma L, Yang DL, Ding WY (2016). Combined effect of 17beta-estradiol and resveratrol against apoptosis induced by interleukin-1beta in rat nucleus pulposus cells via PI3K/Akt/caspase-3 pathway. PeerJ.

[20] Hao XK, Wu W, Wang CX, Xie GB, Li T, Wu HM, Huang LT, Zhou ML, Hang CH, Shi JX (2014). Ghrelin alleviates early brain injury after subarachnoid hemorrhage via the PI3K/Akt signaling pathway. Brain Res.

[21] Waseem T, Duxbury M, Ito H, Ashley SW, Robinson MK (2008). Exogenous ghrelin modulates release of pro-inflammatory and anti-inflammatory cytokines in LPS-stimulated macrophages through distinct signaling pathways. Surgery.

[22] Li JK, Nie L, Zhao YP, Zhang YQ, Wang X, Wang SS, Liu Y, Zhao H, Cheng L (2016). IL-17 mediates inflammatory reactions via p38/c-Fos and JNK/c-Jun activation in an AP-1-dependent manner in human nucleus pulposus cells. J Transl Med.

[23] Endres M, Abbushi A, Thomale UW, Cabraja M, Kroppenstedt SN, Morawietz L, Casalis PA, Zenclussen ML, Lemke AJ, Horn P, Kaps C, Woiciechowsky C (2010). Intervertebral disc regeneration after implantation of a cell-free bioresorbable implant in a rabbit disc degeneration model. Biomaterials.

[24] Koenders MI, Lubberts E, Oppers-Walgreen B, van den Bersselaar L, Helsen MM, Kolls JK, Joosten LA, van den Berg WB (2005). Induction of cartilage damage by overexpression of T cell interleukin-17A in experimental arthritis in mice deficient in interleukin-1. Arthritis Rheum.

[25] Li W, Zhao Y, Xu X, Ma W, Gao P, Wang Y, Liang K, Li R (2015). Rebamipide suppresses TNF-alpha mediated inflammation in vitro and attenuates the severity of dermatitis in mice. FEBS J.

[26] Pfirrmann CW, Metzdorf A, Zanetti M, Hodler J, Boos N (2001). Magnetic resonance classification of lumbar intervertebral disc degeneration. Spine.

[27] Turgut B, Gul FC, Dagli F, Ilhan N, Ozgen M (2013). Impact of ghrelin on vitreous cytokine levels in an experimental uveitis model. Drug Des Devel Ther.

[28] Li L, Zhu L, Hao B, Gao W, Wang Q, Li K, Wang M, Huang M, Liu Z, Yang Q, Li X, Zhong Z, Huang W (2017). iNOS-derived nitric oxide promotes glycolysis by inducing pyruvate kinase M2 nuclear translocation in ovarian cancer. Oncotarget.

[29] Heemskerk S, Masereeuw R, Russel FG, Pickkers P (2009). Selective iNOS inhibition for the treatment of sepsis-induced acute kidney injury. Nat Rev Nephrol.

[30] Croft M, Siegel RM (2017). Beyond TNF: TNF superfamily cytokines as targets for the treatment of rheumatic diseases. Nat Rev Rheumatol.

[31] Gatzka M (2017). Skin under Tnf influence: how regulatory T cells work against macrophages in psoriasis. J Pathol.

[32] Zhang Y, Zhao Y, Li J, Wang S, Liu Y, Nie L, Cheng L (2016). Interleukin-9 promotes TNF-alpha and PGE2 release in human degenerated intervertebral disc tissues. Spine.

[33] Li P, Gan Y, Xu Y, Song L, Wang L, Ouyang B, Zhang C, Zhou Q (2017). The inflammatory cytokine TNF-alpha promotes the premature senescence of rat nucleus pulposus cells via the PI3K/Akt signaling pathway. Sci Rep.

[34] Liu C, Hao Y, Huang J, Li H, Yang Z, Zeng Y, Liu J, Li R (2017). Ghrelin accelerates wound healing in combined radiation and wound injury in mice. Exp Dermatol.

[35] Mwale F, Masuda K, Pichika R, Epure LM, Yoshikawa T, Hemmad A, Roughley PJ, Antoniou J (2011). The efficacy of Link N as a mediator of repair in a rabbit model of intervertebral disc degeneration. Arthritis Res Ther.

[36] Cheng CC, Uchiyama Y, Hiyama A, Gajghate S, Shapiro IM, Risbud MV (2009). PI3K/AKT regulates aggrecan gene expression by modulating Sox9 expression and activity in nucleus pulposus cells of the intervertebral disc. J Cell Physiol.

[37] Edwards A, Abizaid A (2017). Clarifying the ghrelin system's ability to regulate feeding behaviours despite enigmatic spatial separation of the GHSR and its endogenous ligand. Int J Mol Sci.

[38] Abegg K, Bernasconi L, Hutter M, Whiting L, Pietra C, Giuliano C, Lutz TA, Riediger T (2017). Ghrelin receptor inverse agonists as a novel therapeutic approach against obesity-related metabolic disease. Diabetes Obes Metab.

[39] Castro-Mateos I, Hua R, Pozo JM, Lazary A, Frangi AF (2016). Intervertebral disc classification by its degree of degeneration from T2-weighted magnetic resonance images. Eur Spine J.

[40] McCann MR, Seguin CA (2016). Notochord cells in intervertebral disc development and degeneration. J Dev Biol.

[41] Wu N, Chen J, Liu H, Zhao L, Liu S, Liu J, Su X, Wu W, Cong J, Qiu G, Wu Z (2014). The involvement of ADAMTS-5 genetic polymorphisms in predisposition and diffusion tensor imaging alterations of lumbar disc degeneration. J Orthop Res.

[42] Le Maitre CL, Freemont AJ, Hoyland JA (2006). A preliminary in vitro study into the use of IL-1Ra gene therapy for the inhibition of intervertebral disc degeneration. Int J Exp Pathol.

[43] Lee JM, Song JY, Baek M, Jung HY, Kang H, Han IB, Kwon YD, Shin DE (2011). Interleukin-1beta induces angiogenesis and innervation in human intervertebral disc degeneration. J Orthop Res.

[44] Guo J, Shao M, Lu F, Jiang J, Xia X (2017). Role of Sirt1 plays in nucleus pulposus cells and intervertebral disc degeneration. Spine.

[45] Ye F, Wang H, Zheng Z, He P, Sribastav SS, Wang H, Wang J, Liu H, Leung VY (2017). Role of SHOX2 in the development of intervertebral disc degeneration. J Orthop Res.

[46] Wang X, Wang H, Yang H, Li J, Cai Q, Shapiro IM, Risbud MV (2014). Tumor necrosis factor-alpha- and interleukin-1beta-dependent matrix metalloproteinase-3 expression in nucleus pulposus cells requires cooperative signaling via syndecan 4 and mitogen-activated protein kinase-NF-kappaB axis: implications in inflammatory disc disease. Am J Pathol.

[47] Tian Y, Yuan W, Fujita N, Wang J, Wang H, Shapiro IM, Risbud MV (2013). Inflammatory cytokines associated with degenerative disc disease control aggrecanase-1 (ADAMTS-4) expression in nucleus pulposus cells through MAPK and NF-kappaB. Am J Pathol.

[48] Fujita N, Gogate SS, Chiba K, Toyama Y, Shapiro IM, Risbud MV (2012). Prolyl hydroxylase 3 (PHD3) modulates catabolic effects of tumor necrosis factor-alpha (TNF-alpha) on cells of the nucleus pulposus through co-activation of nuclear factor kappaB (NF-kappaB)/p65 signaling. J Biol Chem.

[49] Tran CM, Schoepflin ZR, Markova DZ, Kepler CK, Anderson DG, Shapiro IM, Risbud MV (2014). CCN2 suppresses catabolic effects of interleukin-1beta through alpha5beta1 and alphaVbeta3 integrins in nucleus pulposus cells: implications in intervertebral disc degeneration. J Biol Chem.

[50] Zhao YP, Tian QY, Liu CJ (2013). Progranulin deficiency exaggerates, whereas progranulin-derived Atsttrin attenuates, severity of dermatitis in mice. FEBS Lett.

[51] Tang W, Lu Y, Tian QY, Zhang Y, Guo FJ, Liu GY, Syed NM, Lai Y, Lin EA, Kong L, Su J, Yin F, Ding AH (2011). The growth factor progranulin binds to TNF receptors and is therapeutic against inflammatory arthritis in mice. Science.

[52] Li Z, Lang G, Karfeld-Sulzer LS, Mader KT, Richards RG, Weber FE, Sammon C, Sacks H, Yayon A, Alini M, Grad S (2017). Heterodimeric BMP-2/7 for nucleus pulposus regeneration-In vitro and ex vivo studies. J Orthop Res.

[53] de Vries SA, Potier E, van Doeselaar M, Meij BP, Tryfonidou MA, Ito K (2015). Conditioned medium derived from notochordal cell-rich nucleus pulposus tissue stimulates matrix production by canine nucleus pulposus cells and bone marrow-derived stromal cells. Tissue Eng Part A.

[54] Feng G, Chen H, Li J, Huang Q, Gupte MJ, Liu H, Song Y, Ge Z (2015). Gene therapy for nucleus pulposus regeneration by heme oxygenase-1 plasmid DNA carried by mixed polyplex micelles with thermo-responsive heterogeneous coronas. Biomaterials.

[55] Lin X, Fang X, Wang Q, Hu Z, Chen K, Shan Z, Chen S, Wang J, Mo J, Ma J, Xu W, Qin A, Fan S (2016). Decellularized allogeneic intervertebral disc: natural biomaterials for regenerating disc degeneration. Oncotarget.

[56] Zhang R (2017). Ghrelin suppresses inflammation in HUVECs by inhibiting ubiquitin-mediated uncoupling protein 2 degradation. Int J Mol Med.

[57] Rajan NE, Bloom O, Maidhof R, Stetson N, Sherry B, Levine M, Chahine NO (2013). Toll-Like Receptor 4 (TLR4) expression and stimulation in a model of intervertebral disc inflammation and degeneration. Spine.

[58] Shen C, Cai GQ, Peng JP, Chen XD (2015). Autophagy protects chondrocytes from glucocorticoids-induced apoptosis via ROS/Akt/FOXO3 signaling. Osteoarthritis Cartilage.

[59] Kurashina T, Dezaki K, Yoshida M, Sukma Rita R, Ito K, Taguchi M, Miura R, Tominaga M, Ishibashi S, Kakei M, Yada T (2015). The beta-cell GHSR and downstream cAMP/TRPM2 signaling account for insulinostatic and glycemic effects of ghrelin. Sci Rep.

[60] Fuente-Martin E, Garcia-Caceres C, Argente-Arizon P, Diaz F, Granado M, Freire-Regatillo A, Castro-Gonzalez D, Ceballos ML, Frago LM, Dickson SL, Argente J, Chowen JA (2016). Ghrelin regulates glucose and glutamate transporters in hypothalamic astrocytes. Sci Rep.

[61] Gomez JL, Cunningham CL, Finn DA, Young EA, Helpenstell LK, Schuette LM, Fidler TL, Kosten TA, Ryabinin AE (2015). Differential effects of ghrelin antagonists on alcohol drinking and reinforcement in mouse and rat models of alcohol dependence. Neuropharmacology.

[62] Pirnik Z, Majercikova Z, Holubova M, Pirnik R, Zelezna B, Maletinska L, Kiss A (2014). Effect of ghrelin receptor agonist and antagonist on the activity of arcuate nucleus tyrosine hydroxylase containing neurons in C57BL/6 male mice exposed to normal or high fat diet. J Physiol Pharmacol.

