# Supplementary material for: Ghrelin protects against nucleus pulposus degeneration through inhibition of NF-κB signaling pathway and activation of Akt signaling pathway
Source: Oncotarget. 2017 Jul 31;8(54):91887–901. doi: 10.18632/oncotarget.19695 (PMC5696149; doi:10.18632/oncotarget.19695)
Supplement: Supplementary file 1 [file oncotarget-08-91887-s001.pdf]

Ghrelin protects against nucleus pulposus degeneration through inhibition of NF-κB signaling pathway and activation of Akt signaling pathway

SUPPLEMENTARY MATERIALS

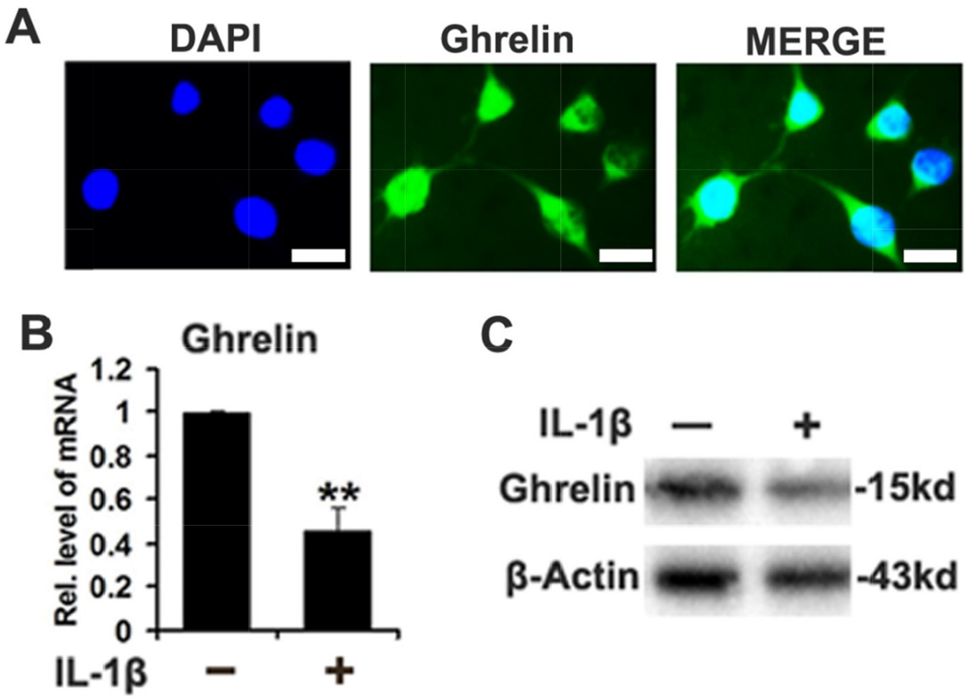

Supplementary Figure 1: Expression pattern of ghrelin in NP cells. (A) Ghrelin is expressed in plasma of primary human NP cells. NP cells were isolated, and cell immunostaining was performed for ghrelin. **(B)** RNA level of ghrelin is diminished by IL-1β, as assessed by real time PCR. NP cells were cultured with 10 ng/ml IL-1β for 12h, and real time PCR was performed for ghrelin. **(C)** Protein level of ghrelin in NP cell is reduced through IL-1β treatment, as assayed by Western blot. The values are the mean±SD. *p<0.05 and ** p<0.01 vs. Control group. Scale bar=25μm. Each experiment was repeated for three times. Scale bar=25μm.

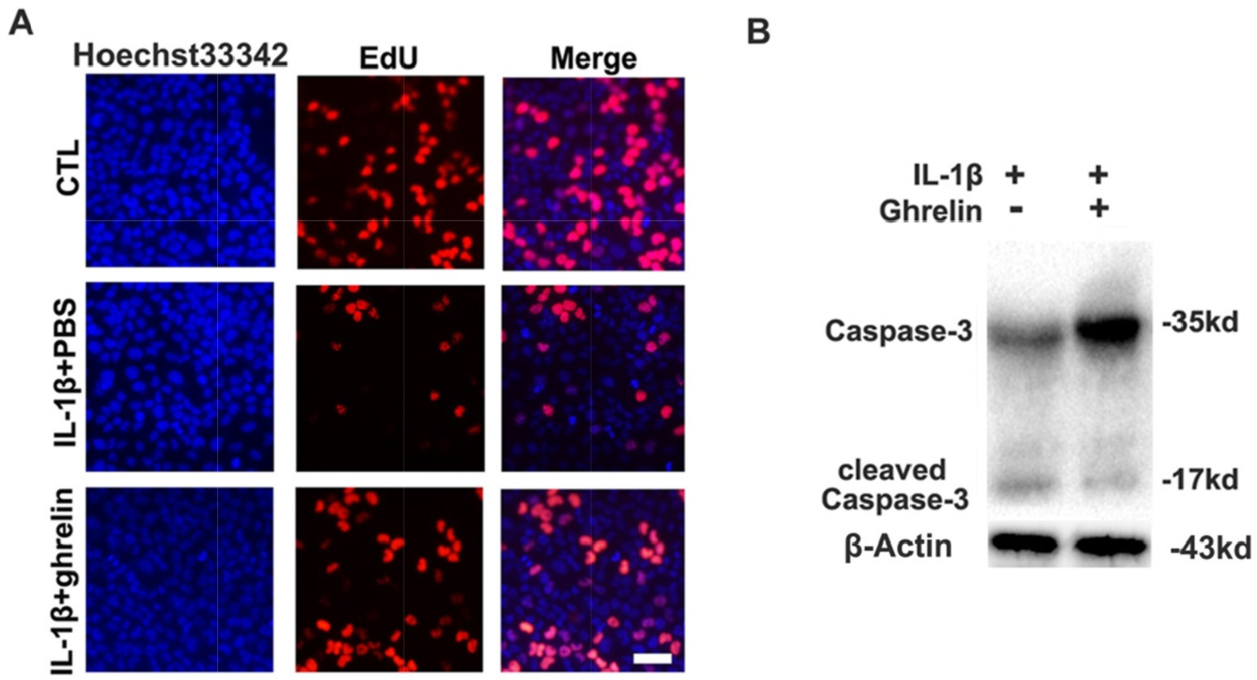

Supplementary Figure 2: Ghrelin promotes proliferation and inhibits apoptosis in IL-1β treated NP cells. (A) The cellular proliferation was detected by EdU proliferation assay. IL-1β treatment diminished the ratio of proliferative cells, while ghrelin reversed cell proliferation in NP cells. Blue dots, all cells; red dots, proliferative cells. **(B)** Western blot analysis of Caspase-3 and Caspase-3 cleavage in NP cells. Primary NP cells were isolated and stimulated with 10n/ml IL-1β for 12 hours, in presence or absence of 50 nM ghrelin, and then Western blot was performed.

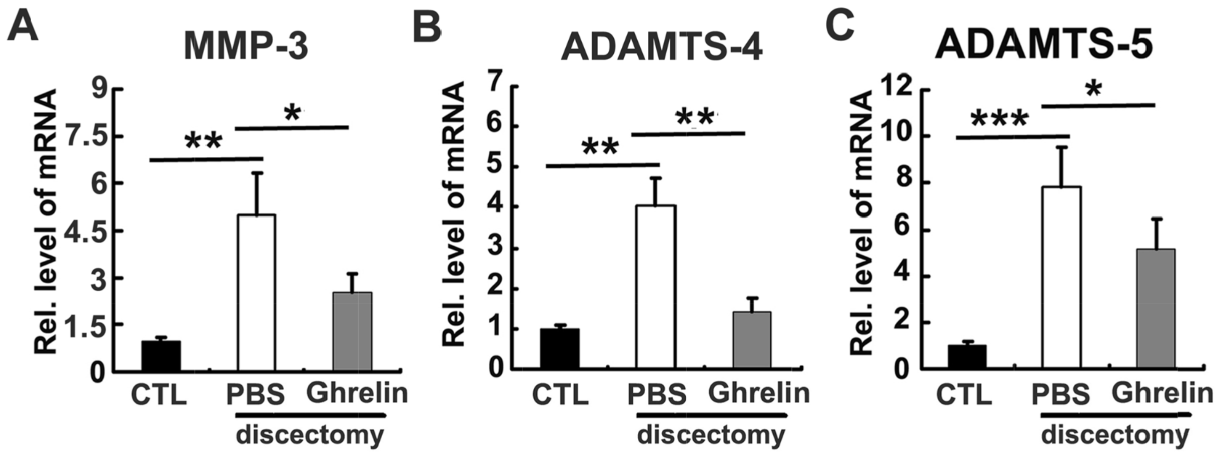

Supplementary Figure 3: Ghrelin protects against catabolic biomarkers of NP tissue in a rabbit IVD degeneration model. (A-C) Ghrelin treatment attenuated the induction of MMP-3, ADAMTS-4 as well as ADAMTS-5 in NP tissue of each treatment group, as assayed by real time PCR. NP tissue was collected and real time PCR was performed. The values are the mean±SD. *$p<0.05$, ** $p<0.01$ and *** $p<0.005$ vs. Control group. Each experiment was repeated for 3 times.
